# Non-Polio Enteroviruses Isolated by Acute Flaccid Paralysis Surveillance Laboratories in the Russian Federation in 1998–2021: Distinct Epidemiological Features of Types

**DOI:** 10.3390/v16010135

**Published:** 2024-01-18

**Authors:** Olga E. Ivanova, Tatiana P. Eremeeva, Nadezhda S. Morozova, Yulia M. Mikhailova, Liubov I. Kozlovskaya, Olga Y. Baikova, Armen K. Shakaryan, Alexandr Y. Krasota, Ekaterina A. Korotkova, Elizaveta V. Yakovchuk, Elena Y. Shustova, Alexander N. Lukashev

**Affiliations:** 1 Federal State Autonomous Scientific Institution “Chumakov Federal Center for Research and Development of Immune-and-Biological Products of the Russian Academy of Sciences” (Institute of Poliomyelitis) (FSASI “Chumakov FSC R&D IBP RAS”), 108819 Moscow, Russiayakovchuklisa@gmail.com (E.V.Y.); shustova_eu@chumakovs.su (E.Y.S.); 2 Department of Organization and Technology of Production of Immunobiological Preparations, Institute for Translational Medicine and Biotechnology, First Moscow State Medical University (Sechenov University), 119048 Moscow, Russia; 3 The Federal Budgetary Health Institution “Federal Center of Hygiene and Epidemiology” of the Federal Office for Inspectorate in the Field of Customers and Human Well-Being Protection”(FBHI FCH&E), 117105 Moscow, Russia; 4 Department of Childrenʹs Infectious Diseases, Pediatric Faculty, Pirogov Russian National Research Medical University, 119121 Moscow, Russia; 5 Belozersky Institute of Physical-Chemical Biology, Lomonosov Moscow State University, 119899 Moscow, Russia; 6 Martsinovsky Institute of Medical Parasitology, Tropical and Vector-Borne Diseases, First Moscow State Medical University (Sechenov University), 119048 Moscow, Russia; 7 Research Institute for Systems Biology and Medicine, 117246 Moscow, Russia

**Keywords:** acute flaccid paralysis (AFP), polioviruses, non-polio enteroviruses, adenoviruses, sewage

## Abstract

More than 100 types of non-polio enteroviruses (NPEVs) are ubiquitous in the human population and cause a variety of symptoms ranging from very mild to meningitis and acute flaccid paralysis (AFP). Much of the information regarding diverse pathogenic properties of NPEVs comes from the surveillance of poliovirus, which also yields NPEV. The analysis of 265 NPEV isolations from 10,433 AFP cases over 24 years of surveillance and more than 2500 NPEV findings in patients without severe neurological lesions suggests that types EV-A71, E13, and E25 were significantly associated with AFP. EV-A71 was also significantly more common among AFP patients who had fever at the onset and residual paralysis compared to all AFP cases. In addition, a significant disparity was noticed between types that were common in humans (CV-A2, CVA9, EV-A71, E9, and E30) or in sewage (CVA7, E3, E7, E11, E12, and E19). Therefore, there is significant evidence of non-polio viruses being implicated in severe neurological lesions, but further multicenter studies using uniform methodology are needed for a definitive conclusion.

## 1. Introduction

Poliovirus (PV) has been the major healthcare concern in the 20th century, and although it is currently under control in most countries, there is a constant threat of re-emergence due to relaxed vaccination policies and persistent circulation of vaccine-derived viruses in many and wild-type viruses in a few countries. PVs belong to the genus *Enterovirus*, and are just three of over more than enterovirus types that infect humans [1]. Thus, PV monitoring largely overlaps with non-polio enterovirus (NPEV) surveillance. NPEVs are ubiquitous and generally replicate in the gut asymptomatically, but may cause a wide spectrum of diseases, primarily in children. They are the main cause of aseptic meningitis, and a few, of which the most noteworthy is enterovirus A71 (EV-A71), are known to cause potentially fatal encephalomyelitis [2]. Also, enteroviruses are a major cause of emerging infections, because new lineages emerge, become dominant, and vanish regularly [3,4,5,6,7], and some of them may cause novel severe clinical manifestations [8,9,10,11].

Poliovirus infection may manifest with acute flaccid paralysis (AFP). Surveillance for this syndrome is thus the key element of the World Health Organization (WHO) Global Polio Eradication Initiative (GPEI) [12,13]. At the same time, AFP can also be a manifestation of infection caused by other viruses or by bacteria, or may have a non-infectious etiology. Guillain–Barré syndrome is the cause of about a half of AFP cases, and polioviruses are associated with just a minor fraction [14]. The laboratory algorithm for the investigation of AFP cases, adopted by the GPEI, includes collection of stool samples from a patient, and isolation and identification of the virus in cell culture [15]. This approach yields as a by-product other cytopathic viruses that are excreted with feces, including NPEVs, human adenoviruses (HAdVs), and human parechoviruses (HPeVs). In most cases, they are not associated with neurological manifestations, and such a connection, if it exists, is very hard to prove, because these viruses are ubiquitous and can be found in healthy people at about the same rate. However, certain types are known or suspected to be more likely than others to cause neurological lesions. A meta-analysis performed by Suresh S. et al. [16] showed that EV-A71, echovirus (E) 13, and E11 were isolated from AFP cases more often, but the etiological role of NPEV in AFP syndrome has been definitively established only for EV-A71 and EV-D68 [16,17,18].

In the Russian Federation (Russia), routine AFP surveillance was introduced in 1998. AFP surveillance is a minimal, but not a comprehensive, approach to poliovirus surveillance because it has limited sensitivity, as only about one in 100 poliovirus infection cases results in AFP. Some countries, including Russia, rely on enterovirus surveillance based on monitoring of compatible clinical cases and/or wastewater surveillance (environmental surveillance), which is also able to detect polioviruses “silently” circulating in the population as an additional methods of poliovirus surveillance [19,20,21,22,23,24]. Systematic surveillance for enterovirus infections (EVIs) in Russia has been gradually expanding since 2009; wastewater studies to monitor PV circulation were carried out optionally by 85 virological laboratories of the Federal Service for Surveillance on Consumer Rights Protection and Human Welfare (Rospotrebnadzor). These latter two approaches were considered as a supplementary surveillance of PV circulation. Therefore, not only PVs, but also NPEVs, isolated in different virological laboratories of the country, were submitted to the National Reference Laboratory for Poliomyelitis (NL) at the M.P. Chumakov Institute of Poliomyelitis and Viral Encephalitides RAS (now the FSASI “Chumakov FSC R&D IBP RAS”) for identification. Thus, significant data on NPEVs circulating in the country between 1998 and 2021 have been accumulated. This paper presents an overview of viruses isolated by the poliomyelitis surveillance network, possible indications of increased capacity of certain types to cause neurological manifestations, dissimilar circulation profiles of enterovirus types, and implications for organizing enterovirus surveillance.

## 2. Materials and Methods

### 2.1. Investigation of AFP Cases in the Russian Federation 

Virological investigation of samples from AFP cases in Russia was carried out by the laboratory network for poliomyelitis surveillance, which consists of six Sub-National Laboratories (SNLs) in the cities of Moscow, St. Petersburg, Yekaterinburg, Omsk, Stavropol, and Khabarovsk and the NL at the FSASI “Chumakov FSC R&D IBP RAS”, Moscow, accredited by the WHO. The NL and SNLs perform investigation of stool samples from AFP cases from their assigned regions in accordance with the WHO guidelines [15] in RD, L20B and, optionally, HEp2c cell cultures. All virus isolates from specimens collected from AFP cases (both polio and non-polio viruses (NPVs)) were transferred to the NL for identification. A total of 10,433 AFP cases were investigated by the Russian polio surveillance network between 1998 and 2021.

### 2.2. Investigation of Samples for Supplementary Surveillance for Polio

Collection and primary investigation of samples within the framework of supplementary surveillance for poliomyelitis (investigation of samples from enterovirus infection cases, wastewater, healthy children from “risk groups”) in Russia is carried out by 85 virological laboratories of the Rospotrebnadzor according to national guidelines and the WHO recommendations [15]. All PV isolates obtained from any material were obligatorily delivered for identification to the NL, and NPEV isolates were delivered optionally at the discretion of the regional laboratories.

#### 2.2.1. Materials from Cases of Enterovirus Infection (EVI)

EVI surveillance was introduced in Russia in 2009. Until 2009, fecal samples were collected sporadically, mainly from aseptic meningitis cases, and tested in RD and HEp2c cell cultures. Isolates were optionally delivered to the NL for identification. Since 2009, cases of aseptic meningitis, exanthema, and herpangina have been a subject for mandatory laboratory testing. Between 2009 and 2018, the proportion of cell culture and molecular detection was gradually shifting towards the latter. Molecular surveillance was carried out by other laboratories, and this study does not include these results. 

Detection of enterovirus RNA in samples of feces, oropharyngeal swabs, and contents of vesicles was carried out by RT-PCR using the AmpliSense Enterovirus-FL kit (FBIS CRIE, Moscow, Russia). The pathogens were identified by VP1 sequencing at the three Reference Centers for EVI surveillance and the NL. The criteria for subjecting isolates for sequencing were neither uniform among laboratories nor continuous throughout the study period. A total of 2008 NPEV isolates were received by the NL from EVI surveillance laboratories between 1998 and 2021.

#### 2.2.2. Isolations from the Environment

For wastewater concentration, most regional laboratories in Russia use trap sampling using bags with sorbent [24]. Viruses were isolated in RD, HEp-2, and L20B cell cultures. From 1998 to 2021, 1816 NPEV isolates were submitted to the NL for identification.

#### 2.2.3. Materials Obtained during the Examination of Risk Groups

In accordance with the Russian national regulations [25], “risk groups” for poliomyelitis include children under 5 years of age from families of migrants, nomadic population groups, children arriving from polio endemic or risk countries, and other healthy children according to epidemiological indications (contacts with a polio patient, children from orphanages, etc.). The study of fecal samples was carried out by the laboratories of the national polio network (NL and SNLs) and the virological laboratories of the Rospotrebnadzor. RD and L20B cell lines were used for virus isolation. In the period 1998–2021, 682 NPEV isolates were submitted to the NL.

### 2.3. Identification of NPVs in the NL

NPEV identification was performed using a neutralization assay according to the standard WHO protocol [15] with pooled polyclonal sera (RIVM, Bilthoven, the Netherlands) for identification of 20 *EV-B* types and one parechovirus (pools A–G) and of 30 non-polio enteroviruses of different types—11 *EV-A*, 12 *EV-B*, 5 *EV-C*, 1 *EV-D*, and one parechovirus (pools H–R). Virus isolates that were not identified in the neutralization test (n = 142) were sequenced in the partial VP1 genome region as described previously [26]. 

Adenoviruses were provisionally identified by typical grape-like cytopathic effect (CPE) in HEp2c cell culture and verified by PCR [27]. 

### 2.4. Clinical Classification of AFP Cases

The final clinical classification of AFP cases was established by the National Commission for the Diagnosis of AFP cases taking into account the presence of residual paralysis on the 60th day from the onset of the disease and the results of laboratory investigation, on the basis of epidemiological investigation and information about the clinical presentations of the disease obtained from the case records. AFP cases were classified on the basis of national guidelines [28] and in accordance with the International Classification of Diseases, 10th revision [29]. 

### 2.5. Statistical Methods

The odds of finding a particular virus type in a given source were evaluated using Fisher’s exact test, comparing the number of isolations for a given type in this and other source(s) versus the number of isolations for all other types from this and other source(s). Types that totaled fewer than 20 isolations from all sources in a given analysis (or 10 isolations in the case of AFP vs. healthy groups) were omitted. Multiple comparison corrections were conducted using the Bonferroni test using R environment.

### 2.6. Ethics Statement

Samples were collected as a part of the Russian national program for polio surveillance, and informed consent for samples investigation was collected by the primary hospitals when necessary. According to the national regulations, the use of anonymous samples and data from state epidemiological surveillance does not require informed consent. 

## 3. Results

### 3.1. NPVs Isolated from AFP Cases

**Figure 1:**
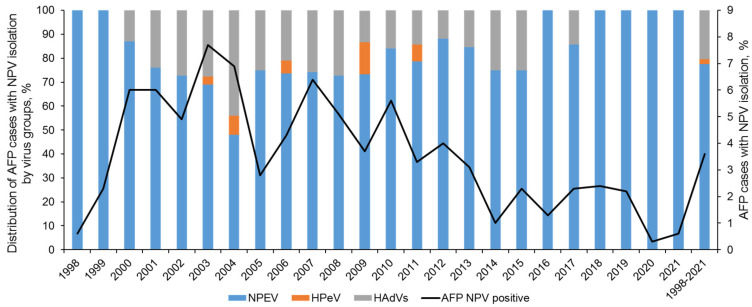
Isolation of non-polio viruses (NPVs) from AFP cases in the Russian Federation in 1998–2021.

Over 24 years of observation in Russia (1998 to 2021), a total of 10,433 AFP cases were investigated, and viruses (cytopathic agents) were isolated from 380 of them (3.6%) (Figure 1). The number of virus-positive cases ranged between 0.3% in 2020 and 7.7% in 2003. Lower virus isolation rates in 1998 can be explained by the unestablished methodology, while in 2020–2021 restriction measures against COVID-19 apparently limited enterovirus circulation. However, even before 2020, there was a clear trend of decreasing virus positivity rates among AFP patients.

Among NPEVs, enterovirus B (EV-B) species were isolated most frequently (221 out of 265, 83.4%), and enterovirus A (EV-A) and C (EV-C) species accounted for 14.7% and 1.9%, respectively (Table 1). Many CVBs were not further identified to type due to lack of clinical and epidemiological indications, and a choice to proceed with identification had been arbitrary. Therefore, the ratio of distinct CVB types is not informative and is provided for reference only. Adenoviruses were not typed systematically. However, in a sample set that largely overlaps with the one reported here, HAdV-C constituted 85% of isolates [27]. 

**Figure 2:**
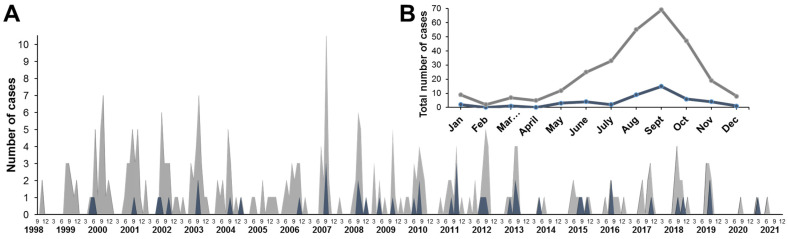
Seasonal distribution of AFP cases with NPEV shedding (gray) and with NPEV shedding, fever at onset, and residual paralysis (blue): (**A**) AFP cases distribution by month from 1998 to 2021; (**B**) total monthly distribution of AFP cases with NPEV isolation from 1998 to 2021. Gray—total number of cases, blue—cases with fever and residual paralysis (60 days after onset) clinically compatible with paralytic forms of poliovirus infection.

Seasonal distribution of AFP cases with NPEV isolations and known date of the disease onset (n = 291; exact onset dates were missing for some samples due to reporting gaps) is shown in Figure 2 (in gray). An increase in the number of cases was observed in the summer–autumn months with a peak in September. We identified a group of AFP cases with fever at the debut of the disease and residual paralysis at least 2 months from the onset of the disease (n = 47), which were clinically compatible with paralytic forms of poliovirus infection (Figure 2, in blue). The annual pattern of these cases corresponded to the overall NPEV isolation pattern; the maximum number of cases was recorded in September (n = 15) (Figure 2B). 

**Figure 3:**
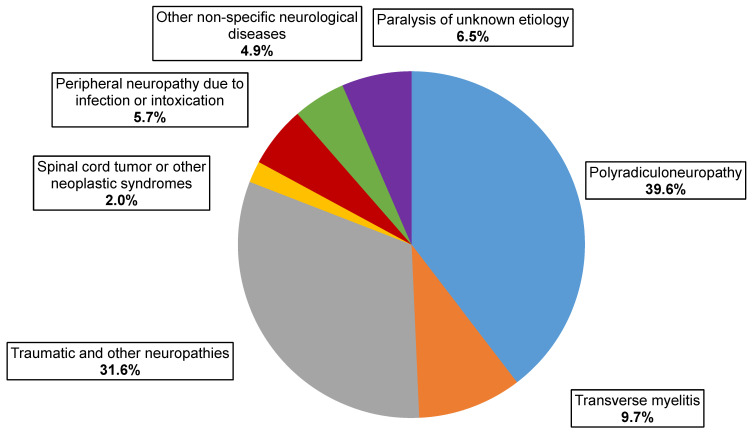
Clinical classification of AFP cases with NPVs isolation.

Final diagnosis data were available for 247 AFP cases, yielding CPE agents that were investigated by the National Commission for Diagnosis Poliomyelitis and AFP. The distribution of AFP cases with NPV isolation by clinical diagnosis showed that the most common were polyradiculoneuropathy, including Guillain–Barré syndrome (98 cases, 39.6%), mononeuropathies, including traumatic ones (78 cases, 31.6%), and myelitis (24 cases, 9.7%) (Figure 3). The same ratio of clinical diagnoses was observed over the same period among all cases of AFP according to the State Sanitary and Epidemiological Surveillance (Rospotrebnadzor)—41%, 32.9%, and 8.8%, respectively. 

The spectrum of viruses isolated from AFP cases with residual paralysis two months after the disease onset is presented in Table 2.

Among 99 cases with residual paralysis, virus types were identified in 91 cases. Viruses most frequently isolated from cases with residual paralysis included CVB1-6 (23 of 91 cases, 25.3%), HAdVs (22 of 91, 24.2%), and EV-A71 (8 of 91, 8, 8%). Among the cases with residual paralysis, 55 had fever at the onset of the disease (out of 59, as 4 cases from which viruses were not identified were excluded); the viruses that were most often isolated from these cases were distributed as follows: HAdVs—25.5% (14 of 55), CVB1-6 group—23.6% (13 out of 55), and EV-A71—12.7% (7 out of 55).

There were no significant differences between frequencies of particular types among AFP cases with residual paralysis and fever and among all AFP. Only EV-A71 was more commonly isolated from AFP cases with residual paralysis and fever at the onset (7 of 55 cases)compared to AFP cases without these two symptoms (7 of 203 cases) (*p* = 0.01).

The role of neurovirulent NPEVs (primarily EV-A71 and EV-D68) in the etiology of myelitis is well known, and the clinical presentation might often be indistinguishable from poliomyelitis caused by poliovirus. Therefore, we separately analyzed myelitis AFP cases, as well as a group of cases of paralysis of unknown etiology. Among 18 myelitis patients with residual paralysis (Table 2), distribution of cytopathogenic agents was as follows: CVB1-6 and HAdVs (4 cases or 22.2% each), CVA2 (3 cases, 16.7%), EV-A71 (2 cases, 11.1%), and CVA16, E3, E6, E21, and E25 (1 case or 5.6% each). Previously, we had described cases of the disease with a clinical picture of poliomyelitis, for which the etiological role of CVA2 was suggested [30]. There were several more noteworthy cases among myelitis patients. Myelitis in a 12-year-old boy fully vaccinated against poliovirus (5 doses of the vaccine) manifested with fever and progressed to a fatal outcome; E11 was isolated from feces. Another case in a 10-year-old unvaccinated child with fever, myelitis, and residual paralysis was associated with CVA16 isolated from the cerebrospinal fluid. The small number of patients with myelitis precludes statistical analysis, but it should be noted that of the few types associated with myelitis, several (EV-A71, E6, E25) were also more common in AFP patients in general compared to children without AFP (Table 1, discussed below). 

In the group “paralysis of unknown etiology”, viruses were identified in three cases only (Table 2): CVA16, EV-A71, and E7. All the cases manifested with a fever at the onset. The child from whom E7 was isolated was not vaccinated against polio; the children with CVA16 and EV-A71 isolations received five and four doses of poliovirus vaccine, respectively.

### 3.2. NPVs Isolated from AFP and Other Sources

The association of certain virus types with AFP was investigated. Most viruses in our dataset (Table 1) were isolated from sewage (n = 1485), followed by healthy subjects (n = 682), and AFP patients (n = 265). There was also a dataset of 1829 isolations from cases of “enterovirus infection” (EVI), arbitrarily identified by local physicians and regional surveillance laboratories. There is no standard definition of EVI, so these cases could include fever, common cold-like symptoms, exanthema, gastroenteritis, meningitis, and other symptoms, but strictly excluding AFP. Isolates from outbreak investigations were excluded from the dataset to reduce sampling bias. 

The distribution of NPEV isolates by enterovirus species from other sources did not differ significantly from the isolations from AFP patients, with the exception of the EV-A species, which was significantly less common in sewage than in other sources (Table 1, *p* < 0.0001, Fisher’s test, Bonferroni correction).

Certain NPEV types were more common in specific sources. The odds of finding each particular virus in a given source (AFP patients, healthy subjects, or sewage) were compared. The ambitious goal was to elucidate the role of NPEV in the occurrence of diseases with persistent neurological lesions similar to poliomyelitis. Therefore, the frequencies of virus isolation from AFP patients were compared with the frequencies of finding these viruses in healthy people as viruses circulating in the population, but not causing disease manifestations. EV-A71 was the only NPEV that was notably more common in AFP patients than in healthy subjects: 56% of all EV-A71 isolations came from AFP compared to 28% of total NPEV isolations from AFP in the dataset. However, this finding was not statistically significant upon Bonferroni correction (*p* = 0.06).

Healthy subjects are better matched controls for the AFP cases. However, the limited number of isolations from this source limited evaluation of the statistical significance of the potential role of other viruses potentially associated with AFP. Thus, we also compared incidence of distinct types among AFP patients to all non-AFP human isolations (healthy and EVI). Three NPEV types were significantly more frequently found in AFP patients than in humans without AFP upon Fisher’s test with Bonferroni correction: EV-A71 (25% of isolates originated from AFP cases), E13 (28%), and E25 (25%), compared to 10% of all enteroviruses originating from AFP patients in this extended dataset. Two types (E6 and E9) were significantly less common among AFP patients. 

It is also noteworthy that certain NPEV types were more or less common in sewage compared to human isolations (Table 1). Types that were significantly more often found in sewage than in human samples were CVA7, E3, E7, E11, E12, E19, and E20. On the contrary, CVA4, CVA10, EV71, CVA9, E9, and E30 were significantly less likely to be isolated from sewage. Most prominently, all 117 E9 isolates collected over the 24 years originated from human samples compared to, for example, 214/302 (71%) of E7 samples found in sewage. 

**Figure 4:**
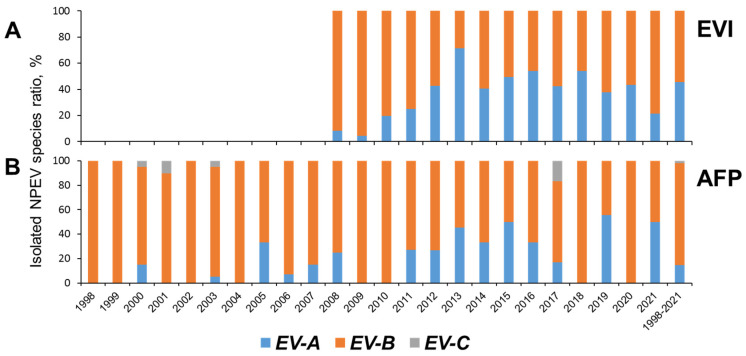
NPEV species isolated from cases of enteroviral infection (EVI, (**A**)) (data from the Reference Center for the Monitoring of Enteroviral Infections in the Federal Budgetary Science Institute “Academician I.N. Blokhina Nizhny Novgorod Scientific Research Institute of Epidemiology and Microbiology”) and acute flaccid paralysis (AFP, (**B**)).

Overall, between 1998 and 2021, EV-B species were the most common among NPEVs obtained by the National Laboratory in Russia, representing 89.6% of isolates, followed by EV-A (8.3%), and EV-C (1.9%). After 2010, the proportion of EV-A steadily began to increase among both AFP and EVI isolates (Figure 4). 

## 4. Discussion

Human enteroviruses (species *Enterovirus A–D*) are ubiquitous, and, along with asymptomatic circulation, can cause diseases with diverse clinical manifestations of varying severity. Human enteroviruses are recognized as a source of emerging diseases because they cause outbreaks, often with a significant public health impact, and sometimes with novel clinical manifestations. Therefore, EV surveillance is an important component of the public health system in many countries. In most countries where EV surveillance is not carried out, AFP surveillance, with clear criteria for sampling and laboratory investigation, is the only source of information on circulating NPEVs. In Russia, EV surveillance has been implemented since 2009, and more comprehensive surveillance results have become available only since 2013. Until then, information about NPEV circulation was obtained irregularly, mainly from EVI outbreaks, primarily aseptic meningitis. Despite the methodological limitations of our study (use of fecal samples as the only clinical material, use of a limited target set of cell cultures for virus isolation, lack of systematic use of molecular methods), this is the most comprehensive study of NPEVs circulating in Russia over a 24-year period: the results of AFP surveillance were supplemented by data from a study of healthy individuals, wastewater, and EVI cases. In addition, the AFP case study methodology was consistent with many other reports obtained from laboratories in the global polio surveillance network (see below).

AFP surveillance is generally not intended to isolate NPEVs. However, the data obtained from this type of surveillance can be compared to results from laboratories in other countries that use the same methodology. During the 24 years of this study, the incidence of NPV isolation ranged from 0.3 to 7.7%, on average 3.6% for any virus and 2.5% for NPEVs. This is significantly lower than the frequency of virus isolation from AFP patients in some countries of Asia and Africa, which could be above 60% [31,32,33], and is comparable or somewhat lower than the frequency determined in some countries of the European Region [34,35]. It is noteworthy that NPEV isolation rates can be used to evaluate the efficiency of surveillance, but the reference regions should be matched in terms of socio-economic and environmental conditions.

The distribution of EV species in our study (Figure 4) was similar to that observed in many countries of the world in different climatic zones according to the results of AFP surveillance [31,32,34,36,37,38], investigation of healthy children [38,39,40], surveillance of EVs [41], and rotavirus surveillance [32]. After 2010, the proportion of EV-A steadily began to increase among both AFP and EVI isolates (Figure 4). A consistently higher share of EV-A among EVI isolates may be a factor due to different sampling (skin lesions and oropharyngeal swabs for EVI) and isolation methodology (strong reliance of AFP surveillance on cell culture that may be suboptimal for EV-A types).

NPEVs have always been “secondary” to polioviruses because they are much less pathogenic and much harder to track and control. Their highly variable pathogenic profile is well known [42]. However, their capacity to cause neurological manifestations stands from all other syn-dromes they can induce and is a major concern. EV-A71, once feared as a “new poliovirus” upon major outbreaks in Bulgaria and Hungary in the 1970s [43], is indeed the most neurovirulent NPEV. However, there have been many hints of other NPEV types being more likely than others to cause central nervous system infections. 

CVB1-6 viruses were the most common among AFP patients (41.1%), followed by E11 (8.3%), E30 (5.7%), E6 and EV-A71 (5.3%), and E25 (4.9%). The same types, with slight variations, were identified as the most common in AFP patients in many studies in countries with different climatic and social conditions [31,34,36,38]. According to two meta-analyses, EV-A71, E13, E11 [16], and E6 [44] were most commonly associated with AFP cases. However, in most studies of this type, there was no proper reference group, making it impossible to establish the role of specific types of NPEVs in AFP onset, because enteroviruses are ubiquitous and most AFP cases have a non-infectious cause. Here, we used healthy subjects and the “EVI” cases as surrogate non-AFP reference groups. Although these were not proper case-control samples and the groups were not uniformly defined, they were collected over the same territory at the same time using a very similar methodology, and the large sample size could offset the potential flaws of the dataset. It is also noteworthy that in the case of enteroviruses and AFP, the utility of precise case-control matches may be limited by asymptomatic circulation of the same EV types that rarely cause AFP. 

EV-A71 emerged as the first AFP suspect in this study, in line with its known neuropathogenic potential. It was significantly more often isolated from AFP patients in general, and significantly more common in AFP patients with fever at the onset and residual paralysis compared to other AFP cases. This finding, though trivial, indicates the fitness of the datasets and the approach used here to identify potentially neurovirulent NPEVs. Type E13, which has been significantly over-represented among AFP patients compared to non-AFP sources here, has also been implicated in several studies as a potential AFP etiological agent [16]. However, only 2 out of 12 E13 isolates from AFP patients came from children with fever at the onset and residual paralysis (typical signs of AFP caused by a poliovirus), a dramatic contrast with 7 of 14 EV-A71 isolations. This suggests that even though there is strong evidence here and in other publications that E13 is associated with neurological lesions, they might often be transient and not as severe as upon poliovirus or EV-A71 infection. E25 was significantly over-represented among AFP patients and relatively common among patients with fever and residual paralysis. Another suspected AFP cause, E11 [16], was also somewhat over-represented in AFP patients in our study; however, the statistical significance of this finding did not pass the Bonferroni correction. Types E6, E9, E18, and E30, each implicated in one or few studies as a potential AFP or encephalitis cause [16], were, on the contrary, under-represented in AFP patients in our study. Despite the robust statistical significance of this finding, it might be premature to make a conclusion about the lower pathogenicity of these types. Alternative explanations could include variation of pathogenic properties between subtypes that were circulating in different countries and at different times, or extensive circulation of these types among age groups that were less likely to have AFP because of unrelated causes. CVA2, which has been implicated in AFP cases in Russia and in Brazil [30,36], has been twice more common among AFP patients than average in the dataset; however, this was not statistically significant due to a small overall number of CVA2 isolations. On the other hand, three out of five CVA2—positive AFP patients had fever at the onset and residual paralysis, comparable with virus-induced poliomyelitis. Thus, CVA2 should remain high in the list of potential NPEVs capable of causing AFP. CVA4 and CVA16 were not more frequent among AFP patients than average; however, a notable fraction of isolations in AFP cases (2/7 and 2/4, respectively) came from patients with fever at the onset and residual paralysis. It is noteworthy that EV-D68, implicated in neurological disease in many studies [6,42], was not found in our study, because this type is predominantly a respiratory virus, and oral swabs have been rarely studied. It is also interesting that adenoviruses represented 14 out of 59 (24%) total virus isolations from patients with fever at the onset and residual paralysis. This was not significantly more often than in all AFP patients (70/342, 20.5%). Thus, adenoviruses were not associated with the polio-like AFP manifesting with fever and residual paralysis. Previous studies, including one carried out on this sample set, reported significantly higher incidence of adenoviruses among all AFP patients compared to healthy children [27,45,46]. Thus, adenoviruses might be linked to other clinical forms of AFP, not polio-like types. Alternatively, adenoviruses could simply be associated with AFP due to a common predisposing factor without a direct causative link. 

Proving the etiological role of ubiquitous and moderately pathogenic viruses, such as enteroviruses and adenoviruses, in poly-etiological diseases can be very challenging. This is best exemplified by type I diabetes, where multiple works found a relatively weak association with enterovirus infection and implied different mechanisms, but a huge body of diverse studies with their own limitations make up rather reliable evidence of the role of NPEVs in pathogenesis [47,48]. Low incidence of AFP in general, and association of most cases with non-virus causes, augments the challenge of investigating the role of infection in etiology. Even a matched case-control sample may have its bias in this case, because, for example, obviously pathogenic enteroviruses, such as poliovirus, cause paralytic disease in just about 1% of patients, and thus may be present in perfectly matched controls that were involved in community circulation of the same viruses. Nevertheless, when multiple studies find a weak association of NPEVs and AFP, this suggests future directions for enterovirus surveillance.

The potential of NPEVs to cause diverse and often emerging diseases, as well as continuing circulation of poliovirus, justify the need for surveillance. There are several approaches to poliovirus and enterovirus surveillance, and each country chooses a combination depending upon the epidemiological situation and available resources. Two principal methods are human (patient or healthy)-based and environment-based surveillance. In the case of poliovirus, AFP surveillance requires a significant effort to establish, but then it is relatively cost-efficient (few samples need to be processed to detect poliovirus). However, as poliovirus causes AFP in roughly 1% of infected children, this approach may miss low-level circulation. Environment (sewage)-based surveillance is easier to establish, but is more expensive in terms of equipment and reagents for high-throughput sequencing. The same considerations apply to NPEV surveillance. However, this choice is complicated by the different prevalence of enteroviruses in healthy and sick humans, and in sewage. This disparity was best demonstrated in a recent study that showed distinct (although overlapping) profiles of enterovirus types in sewage and among clinical isolates [10]. In our dataset that covers 24 years of observation and excludes overt outbreaks, there was also a significant disagreement between the prevalence of EV types in human samples and in sewage. In fact, 16 of 24 types with >20 isolations were significantly over- or under-represented in sewage upon the Bonferroni correction. Predominantly “patient” viruses included CVA2, CVA9, EV-A71, E9, and E30, which were also often reported to cause human disease in other studies. On the contrary, most of the types that were over-represented in sewage (CVA7, E3, E7, E11, E12, and E19) are rarely reported to cause human disease elsewhere. Interestingly, E11, a virus with a well-known pathogenic potential, was also over-represented in sewage in our study. This may be explained by distinct epidemiological profiles of E11 subtypes, some of which are much more likely to be found in sewage than others [49].

Therefore, the circulation profile of certain types is generally compatible in different studies all over the world. It should be noted, however, that the prevalence of a certain enterovirus type in sewage may also be defined by its replication/excretion levels or capsid stability. Nevertheless, differences between the observed circulation profiles of distinct types are stark.

## 5. Conclusions

AFP surveillance has been carried out in the Russian Federation for more than 25 years. The methodology has shown high efficiency in detecting poliomyelitis caused by poliovirus (including vaccine-associated), but is clearly insufficient to obtain substantiated evidence of the role of non-polio enteroviruses in the occurrence of AFP. Maintaining AFP surveillance, which has well-defined case-finding criteria, a network of WHO-accredited laboratories, and a computerized database of epidemiological information and virological results, seems a rational solution even considering the forthcoming certification of polio eradication. Surveillance and diagnostic capabilities for investigating the infectious etiology of AFP should be enhanced through the systematic collection of additional material (oropharyngeal swabs, CSF) and the use of molecular methods (PCR and high-throughput sequencing) for the detection and identification of viruses. It is also critical to include non-AFP sources, ideally healthy subjects matched by age, gender, sampling location, and date, and to increase the sample by multicenter international studies. 

Various approaches to monitoring circulating NPEVs in the population (surveillance for AFP, enterovirus surveillance, examination of materials from healthy children, sewage) provide different data. We believe that the most comprehensive epidemiological analysis and prognosis for non-polio enterovirus infections can be obtained by comparing the results of different types of surveillance. This expensive and difficult-to-implement approach can become realistic with the widespread introduction of high-throughput molecular methods of investigation.

## Figures and Tables

**Table 1:** Non-polio enteroviruses isolated from AFP cases and supplementary poliovirus surveillance in the Russian Federation, 1998–2021.

EV Type ^a^	AFP			Healthy ^b^	Enterovirus Infection (EVI) ^c^		Sewage	
	Isolates (%)	vs. Healthy ^d^	vs. Healthy + EVI	Isolates (%)	Isolates (%)	vs. Healthy	Isolates (%)	vs. Any Other
CVA2	5 (1.9)	n.s. ^e^	n.s.	9 (1.3)	7 (0.4)	n.s.	0 (0)	<0.01
CVA4	7 (2.6)	n.s.	n.s.	29 (4.3)	26 (1.4)	n.s.	8 (0.5)	n.s.
CVA7	0 (0)	n.s.	n.s.	5 (0.7)	5 (0.3)	n.s.	21 (1.4)	<0.01
CVA10	3 (1.1)	n.s.	n.s.	13 (1.9)	31 (1.7)	n.s.	5 (0.3)	n.s.
CVA16	4 (1.5)	n.s.	n.s.	2 (0.3)	47 (2.6)	<0.01	25 (1.7)	n.s.
EV-A71	14 (5.3)	n.s.	<0.05	11 (1.6)	32 (1.7)	n.s.	3 (0.2)	<0.001
**Total *EV-A***	**39**	**–**	**–**	**73**	**172**	**–**	**69**	**–**
CVA9	2 (0.8)	n.s.	n.s.	2 (0.3)	50 (2.7)	<0.001	1 (0.1)	<0.001
CVB1-6	109 (41.1)	n.s.	n.s.	247 (36.2)	421 (23.0)	n.s.	538 (36.2)	<0.001
E3	11 (4.2)	n.s.	n.s.	16 (2.3)	16 (0.9)	n.s.	46 (3.1)	<0.05
E4	0 (0)	n.s.	n.s.	4 (0.6)	21 (1.1)	n.s.	15 (1.0)	n.s.
E6	14 (5.3)	n.s.	<0.05	42 (6.2)	264 (14.4)	<0.001	157 (10.6)	n.s.
E7	9 (3.4)	n.s.	n.s.	15 (2.2)	64 (3.5)	n.s.	214 (14.4)	<0.001
E9	2 (0.8)	n.s.	<0.05	30 (4.4)	85 (4.6)	n.s.	0 (0)	<0.001
E11	22 (8.3)	n.s.	n.s.	46 (6.7)	70 (3.8)	n.s.	170 (11.4)	<0.001
E12	1 (0.4)	n.s.	n.s.	7 (1.0)	9 (0.5)	n.s.	41 (2.8)	<0.001
E13	12 (4.5)	n.s.	<0.05	23 (3.4)	8 (0.4)	<0.001	11 (0.7)	n.s.
E14	6 (2.3)	n.s.	n.s.	13 (1.9)	11 (0.6)	n.s.	5 (0.3)	n.s.
E17	0 (0)	n.s.	n.s.	18 2.6)	7 (0.4)	<0.001	14 (0.9)	n.s.
E19	3 (1.1)	n.s.	n.s.	16 (2.3)	17 (0.9)	n.s.	56 (3.8)	<0.001
E25	13 (4.9)	n.s.	<0.05	16 (2.3)	24 (1.3)	n.s.	33 (2.2)	n.s.
E29	0 (0)	n.s.	n.s.	15 (2.2)	2 (0.1)	<0.001	13 (0.9)	n.s.
E30	15 (5.7)	n.s.	n.s.	26 (3.8)	540 (29.5)	<0.001	41 (2.8)	<0.001
**Total *EV-B***	**22** **1**	**–**	**–**	**579**	**1634**	**–**	**1384**	**–**
CVA21	3 (1.1)	n.s.	n.s.	2 (0.3)	12 (0.7)	n.s.	6 (0.4)	
CVA24	2 (0.8)	n.s.	n.s.	23 (3.4)	10 (0.5)	<0.001	23 (1.5)	
**Total *EV-C***	**5**	**–**	**–**	**30**	**23**	**–**	**32**	**–**
**All types**	**265**	**–**	**–**	**682**	**1829**	**–**	**1485**	**–**

^a^ Types with fewer than 20 isolates in total were omitted from the table, but were included in statistical analysis. ^b^ The “Healthy” group includes isolates from the risk groups (children from migrant families and nomadic populations, children arriving from polio endemic and risk countries) and healthy children upon epidemiological indications (contacts with AFP cases, children from orphanages). ^c^ “Enterovirus infection” was a diagnosis at sample submission as defined by the field laboratories, and included cases of meningitis (excluding outbreaks), HFMD, herpangina, gastroenteritis, influenza-like disease, etc., but excluded AFP. ^d^ Fisher’s exact test of odds with Bonferroni multiple comparison correction was used. ^e^ n.s., not statistically significant.

**Table 2:** Non-polio viruses isolated from AFP cases with residual paralysis; cases that additionally had fever at the disease onset are shown in parentheses.

NPVs Cases with Residual Paralysis (Cases with Residual Paralysis and Fever)			Final Classification of AFP Cases According to ICD 10 ^a^							
			2 n = 34 (18)	3 n = 20 (13)	4 n = 33 (19)	5 n = 4 (1)	6 n = 2 (2)	7 n = 3 (3)	9 n = 3 (3)	Total n = 99 (59)
EVs n = 66 (40)	*EV-A* n = 15 (14)	CVA2	– ^b^	3 (3)	–	–	–	–	–	3 (3)
		CVA4	–	–	1 (1)	–	–	1 (1)		2 (2)
		CVA16	–	1 (1)		–	–		1 (1)	2 (2)
		EV-A71	2 (2)	2 (2)	2 (1)	–	–	1 (1)	1 (1)	8 (7)
	*EV-B* n = 51 (26)	CVA9	–	–	2	–	–	–	–	2
		CVB1-6	9(5)	4 (3)	8 (4)	1	–	1 (1)	–	23 (13)
		E3	1	1	–	1	–	–	–	3
		E6	–	1	–	1	–	–	–	2
		E7	–	–	1	–	–	–	1 (1)	2 (1)
		E9	–	–	1 (1)	–	–	–	–	1 (1)
		E11	3 (2)	–	1 (1)	–	–	–	–	4 (3)
		E13	1 (1)	–	1 (1)	–	–	–	–	2 (2)
		E14	1	–	–	–	–	–	–	1
		E21	–	1 (1)	–	–	–	–	–	1 (1)
		E25	1	1 (1)	1 (1)	–	1 (1)	–	–	4 (3)
		E30	4 (2)	–	1	–		–	–	5 (2)
		E33	1	–	–	–	–	–	–	1
NTVs			4 (2)	2 (1)	2 (1)	–	–	–	–	8 (4)
HAdVs			5 (3)	4 (1)	11 (8)	1 (1)	1 (1)	–	–	22 (14)
HPeVs			2 (1)	–	1	–	–	–	–	3 (1)

^a^ ICD 10—International Statistical Classification of Diseases and Related Health Problems 10th Revision: 2—polyradiculoneuropathy; 3—transverse myelitis; 4—traumatic neuropathies, other mononeuropathies; 5—spinal cord tumor, other neoplasms; 6—peripheral neuropathy due to infection or intoxication; 7—other nonspecific neurological diseases; 9—paralysis of unknown etiology. ^b^—no isolations.

## Data Availability

The data presented in this study are available in this article.
